# Striatal Atrophy in the Behavioural Variant of Frontotemporal Dementia: Correlation with Diagnosis, Negative Symptoms and Disease Severity

**DOI:** 10.1371/journal.pone.0129692

**Published:** 2015-06-15

**Authors:** Matthew D. Macfarlane, David Jakabek, Mark Walterfang, Susanna Vestberg, Dennis Velakoulis, Fiona A. Wilkes, Christer Nilsson, Danielle van Westen, Jeffrey C. L. Looi, Alexander Frizell Santillo

**Affiliations:** 1 Illawarra Shoalhaven Local Health District, Wollongong, Australia; 2 Graduate School of Medicine, University of Wollongong, Wollongong, Australia; 3 Melbourne Neuropsychiatry Centre, Royal Melbourne Hospital, Melbourne, Australia; 4 University of Melbourne, Melbourne, Australia; 5 Department of Psychology, Lund University, Lund, Sweden; 6 Research Centre for the Neurosciences of Ageing, Academic Unit of Psychiatry and Addiction Medicine, School of Clinical Medicine, Australian National University Medical School, Canberra, Australia; 7 Clinical Memory Research Unit, Department of Clinical Sciences, Lund University, Lund, Sweden; 8 Center for Medical Imaging and Physiology, Skåne University Hospital, Lund, Sweden; 9 Diagnostic Radiology, Department of Clinical Sciences, Lund University, Lund, Sweden; 10 Geriatric Psychiatry Unit, Department of Clinical Sciences, Lund University, Lund, Sweden; Taipei Veterans General Hospital, TAIWAN

## Abstract

**Introduction:**

Behavioural variant frontotemporal dementia (bvFTD) is associated with changes in dorsal striatal parts of the basal ganglia (caudate nucleus and putamen), related to dysfunction in the cortico-striato-thalamic circuits which help mediate executive and motor functions. We aimed to determine whether the size and shape of striatal structures correlated with diagnosis of bvFTD, and measures of clinical severity, behaviour and cognition.

**Materials and Methods:**

Magnetic resonance imaging scans from 28 patients with bvFTD and 26 healthy controls were manually traced using image analysis software (ITK-SNAP). The resulting 3-D objects underwent volumetric analysis and shape analysis, through spherical harmonic description with point distribution models (SPHARM-PDM). Correlations with size and shape were sought with clinical measures in the bvTFD group, including Frontal Behavioural Inventory, Clinical Dementia Rating for bvFTD, Color Word Interference, Hayling part B and Brixton tests, and Trail-Making Test.

**Results:**

Caudate nuclei and putamina were significantly smaller in the bvFTD group compared to controls (left caudate 16% smaller, partial eta squared 0.173, p=0.003; right caudate 11% smaller, partial eta squared 0.103, p=0.023; left putamen 18% smaller, partial eta squared 0.179, p=0.002; right putamen 12% smaller, partial eta squared 0.081, p=0.045), with global shape deflation in the caudate bilaterally but no localised shape change in putamen. In the bvFTD group, shape deflations on the left, corresponding to afferent connections from dorsolateral prefrontal mediofrontal/anterior cingulate and orbitofrontal cortex, correlated with worsening disease severity. Global shape deflation in the putamen correlated with Frontal Behavioural Inventory scores—higher scoring on negative symptoms was associated with the left putamen, while positive symptoms were associated with the right. Other cognitive tests had poor completion rates.

**Conclusion:**

Behavioural symptoms and severity of bvFTD are correlated with abnormalities in striatal size and shape. This adds to the promise of imaging the striatum as a biomarker in this disease.

## Introduction

Frontotemporal lobar degeneration (FTLD) is the term for a group of neurodegenerative disorders that are characterised in their behavioural variant (bvFTD) by disinhibition, apathy, coarsening of personality, repetitive behaviour, and eating disturbances [1, 2]. The language variants include progressive nonfluent aphasia (PNFA) and semantic dementia (SD), and all variants can overlap with atypical Parkinsonian disorders such as progressive supranuclear palsy and corticobasal degeneration. Our focus in this study is on bvFTD, the most common subtype, and is aimed toward understanding the structural basis of cognitive and behavioural changes seen in this disorder.

**Fig 1:**
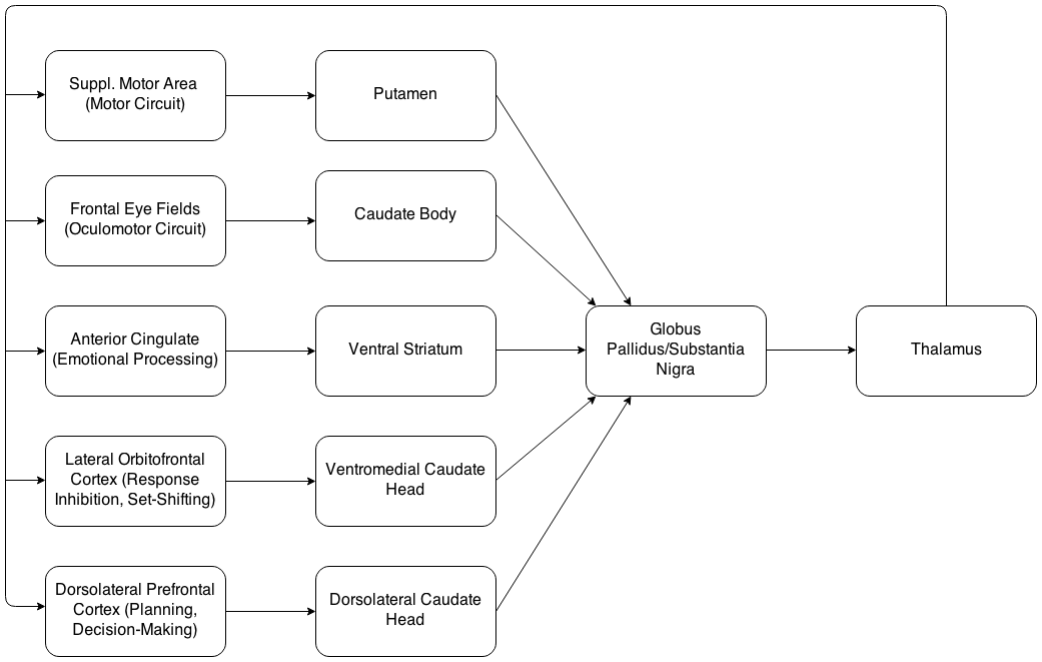
Representation of the cortico-striato-thalamic circuits. Modified from [6].

**Fig 2:**
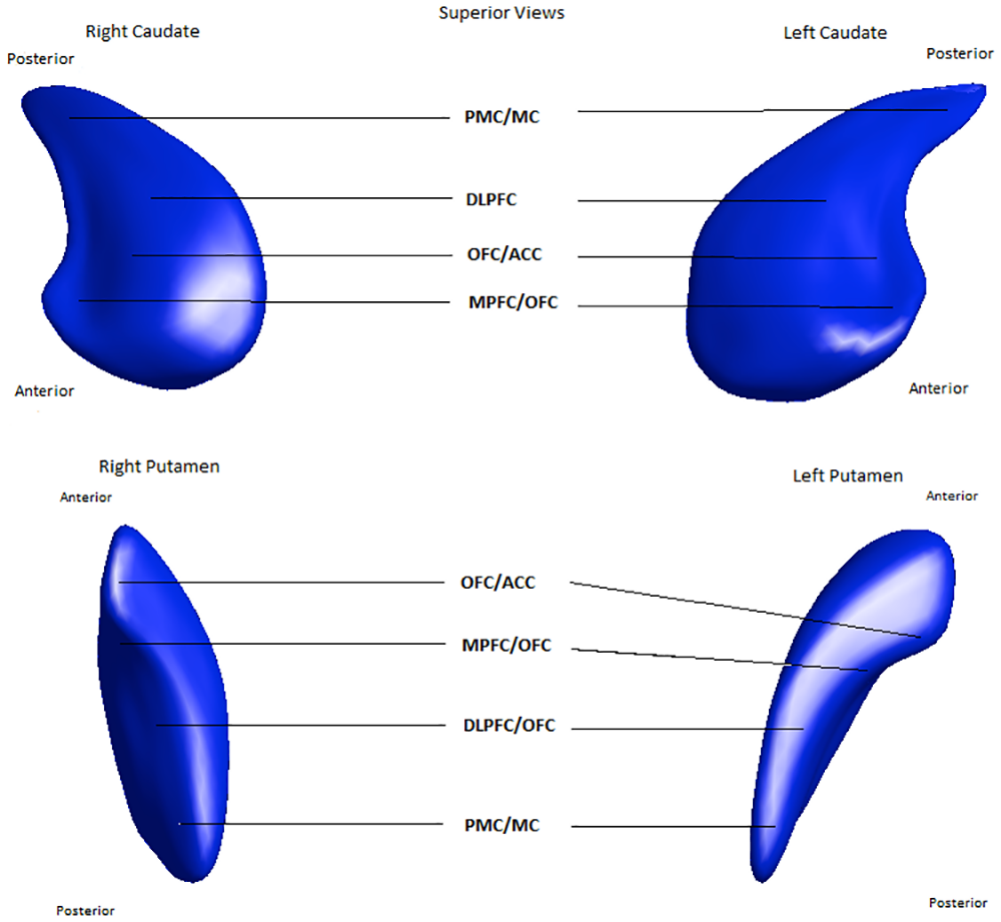
The location of afferent connections on caudate and putamen. ACC = Anterior cingulate cortex, DLPFC = Dorsolateral prefrontal cortex, MC = Motor cortex, MPFC = Medial prefrontal cortex, OFC = Orbitofrontal cortex, PMC = Premotor cortex.

While previous research has focused on cortical changes in the pathophysiology of bvFTD, relevant cortico-striato-thalamic circuits are also implicated [3–5]. These re-entrant circuits run from specific cortical areas to corresponding areas on basal ganglia structures such as the striatum, which connects to the globus pallidus and substantia nigra, before connecting to the thalamus and completing the circuit to the cortex [6] (Figs 1 and 2). Accordingly, in neurodegenerative disorders that are characterised by dysfunction in these circuits, structural and functional changes in different nodes within the circuits are likely, that is, structural change can be seen as a biomarker for functional change [5]. As there is also somatotopic localisation of the cortico-striato-thalamic circuits to the surface and interior of the striatum [4], examining striatal shape deformities in addition to striatal size can potentially pinpoint areas of these structures that have disproportionately atrophied, allowing a finer-grained description of the likely frontostriatal circuits that are affected [7].

Following on from this theoretical work, in addition to recent studies investigating the pattern of cortical changes in bvFTD there have also been a number of studies specifically examining brain changes in subcortical structures in this disease [8]. Change in the striatum has been demonstrated, with caudate nucleus shape differences occurring in a pattern that differs from that of other neurodegenerative disorders [9, 10] and differs within subtypes of FTLD [11]. Similar changes in the putamen have also been demonstrated [9, 12], with striatal changes appearing to be more frequent in tau-positive disorder [13], but disproportionate caudate changes have also been observed in disease characterised by the fused in sarcoma protein (FUS) [14]. Resting state fMRI studies support the concept of a diaschisis between cortical and subcortical structures driving some of the behavioural symptoms of bvFTD and other dementias, showing that specific intrinsic connectivity networks tend to degenerate in cortical and subcortical areas as a group [15, 16]. While the aforementioned studies have demonstrated robust differences between diagnostic groups, there have been few studies looking specifically at striatal size and shape and correlating this with standardised measures of behavioural disturbance and cognitive function in bvFTD.

The striatal structures have been linked with planning and implementation of motor functions, as well as executive functioning, in the normal brain [17], so changes in these structures would be expected to correlate with tests of these cognitive functions. Our group has found measures of executive function to correlate with caudate size in an older adult group with diffuse white-matter changes [18], but the clinical significance of striatal changes in bvFTD are less known. Thus, the goal of this study is to extend the previous findings of our group by attempting to confirm the groupwise shape deformations in striatal structures seen in bvFTD, as well as correlate size and shape of caudate nucleus and putamen (as structural substrates of cortico-striato-thalamic circuits) with measures of behavioural disturbance and cognitive dysfunction in bvFTD.

## Materials and Methods

Participants were from the Lund Prospective Frontotemporal Dementia Study (LUPROFS), a longitudinal study of patients with any of the frontotemporal dementia spectrum disorders [19]. Diagnosis of bvFTD was made at diagnostic conference, according to Frontotemporal Lobe Degeneration Consensus (FTDC) criteria [2]. Workup included clinical examination, caregiver history with rating of behavioural disturbances and disease severity, standardized neurological examination, MRI according to study protocol, CSF analysis of Alzheimer´s Disease (AD) core biomarkers (amyloid-beta-42, tau and p-tau) and a comprehensive neuropsychological assessment. Among the bvFTD patients (*n* = 28), 20 had probable, two possible and six definite bvFTD, according to FTDC criteria. The two individuals with a possible FTD diagnosis did display macroscopic atrophy on MRI, however not solely confined to the frontal and/or temporal lobes. The diagnosis of probable FTD rested on the study MRI in all cases but three, for which results of clinical functional imaging (SPECT or FDG-PET) were used. None of the patients displayed a CSF biomarker profile indicative of AD [20]. Healthy controls (*n* = 26) underwent clinical interview and examination, including neuropsychological testing and MRI. Demographic and clinical data on the subjects are presented in Table 1.

**Table 1:** Demographics and Clinical Factors.

	**Healthy Control**	**bvFTD**	**Comparison**
****Number****	26	28	-
****Sex****	13M 13F	17M 11F	p = 0.599
****Age****	68 (35–82)	71 (38–78)	p = 0.093
****MMSE****	30 (29–30)	25 (12–30)	p = 0.000
****Education (years)****	14 (8–14)	9 (1–15)	p = 0.016
****Handedness (r/l)****	-	27/1	-
****CDR****	n.a.	1 (0–2)	-
****FTLD-CDR-SB****	n.a.	9 (2–16.5)	-
****Duration of illness (years)****	n.a.	3 (1–12)	-
****FBI**^**1-10**^**score****	n.a.	16 (6–29)	-
****FBI**^**12-22**^**score****	n.a.	8 (1–24)	-
****FBI total score****	n.a.	27 (11–58)	-

bvFTD = Behavioural Variant Frontotemporal Dementia, CDR = Clinical Dementia Rating, FBI = Frontal Behavioural Inventory, MMSE = Mini-Mental State Examination, CDR = Clinical Dementia Rating, r/l = right/left handed, FTLD-CDR-SB = Frontotemporal Lobar Degeneration modified Clinical Dementia Rating Sum of Boxes. All values median, (minimum-maximum). Comparisons are with Mann-Whitney test, and Chi-square for sex distribution.

### Ethics Statement

Patients and healthy controls were informed of the study content in both oral and written form. Informed consent was taken in written form from the study subjects. If there was a suspicion that the study subject had a compromised capacity to consent, informed consent was taken from the study subject and the spouse, or the spouse only. In any of these situations, the study subject retained the right to decline or interrupt participation at any time. No formal assessment of the patients capacity to consent was performed, instead this was determined by the clinician. All subjects received a copy of the study information and their informed consent. The procedure for informed consent, as well as all other aspects of the study, was approved by the Regional Ethical Review Board, Lund, Sweden (Permit number 617/2008).

### Cognitive Testing and Behavioural Rating

For a rating of general disease severity we employed the Clinical Dementia Rating (CDR) score, and the sum of boxes in the Frontotemporal Lobar Degeneration modified Clinical Dementia Rating (FTLD modified CDR) (Knopman et al., 2008)—the latter has the advantage of adding two FTLD characteristic items, behaviour and language, to the CDR. Quantification of behaviour disturbances in the bvFTD group were conducted using the Frontal Behavioural Inventory (FBI) (Kertesz, Davidson, & Fox, 1997) which rates 24 behaviours (items) on a 0–3 scale.

For the purpose of this study, the FBI was divided into subscores for FBI items 1–10, which represent negative symptoms such as apathy, emotional flatness, and personal neglect; and FBI items 12–22, representing positive symptoms such as perseverations, excessive jocularity, poor judgement, inappropriateness, impulsivity and hyperorality. This division of composite scales and/or patients is a common procedure in bvFTD studies [21–23]. A higher score on the FBI represents more severe symptomatology.

The following neuropsychological tests were performed on the participants in the bvFTD group as tolerated, chosen on the basis that they focus on executive cognitive function, a key clinical feature of bvFTD considered to be localised to the dorsolateral prefrontal cortico-striato-thalamic circuit traversing the striatum [6, 24, 25]: Color Word Interference from the Delis-Kaplan Executive Function System (D-KEFS) [26]; Hayling part B and Brixton tests [27]; and Trail-making Test B [28, 29].

### MRI Acquisition

MRI was performed using a Philips Achieva 3T scanner equipped with an eight-channel head coil. Subsequent quantitative analysis is based on a T_1_-weighted 3D volumetric sequence with TR 8.3 ms, TE 3.84 ms, FOV 256x256x175 and a voxel size of 1x1x1 mm^3^. T1, FLAIR and T2 images were inspected for the presence of cerebrovascular lesions in the caudate and/or putamen by a senior neuroradiologist (DvW) and those with lesions were excluded from volume and shape analysis.

### Image Analysis

MRI scans were analysed with ITK-SNAP [30], using a standardised and validated manual tracing method for the caudate [31] and putamen [12]. This method produced 3-D object maps of the caudate and putamen, from which volume and shape analysis calculations were made. These tracing delineate these structures in their whole, except the posterior part of the caudate tail, as it curves ventrally. Importantly, the tracing protocols do not include the ventral striatum/nucleus accumbens. Inter-rater reliability was measured by having a second researcher (FAW) trace a representative sample of the subjects—for the caudate, the intraclass correlation coefficient (absolute agreement, average measures) was 0.93, and 0.85 for the putamen. Intra-rater reliability of the researcher performing the tracings (MM) was 0.98 for the caudate and 0.93 for the putamen.

Intracranial volume (ICV) was determined in a semi-automated fashion using FSL software (FMRIB Group, Oxford, Oxfordshire, United Kingdom) as a measure to control for brain size. First, brains were skull-stripped with the Brain Extraction Tool (http://www.fmrib.ox.ac.uk/analysis/research/bet/) and were then linearly aligned to the MNI (Montreal Neurological Institute, McGill University Montreal, Canada) 152 1-mm T1-weighted template [32]. The inverse of the determinant of the affine transformation matrix was multiplied by the ICV of the MNI152 template to produce a measure of ICV for use as a covariate [32, 33].

### Statistical Analysis

Comparisons of demographic data between bvFTD patients and controls were made using Mann-Whitney test (or Pearson Chi-square for gender) in IBM SPSS Statistics Version 21. ANCOVA was performed to determine groupwise differences between the bvFTD and control groups, while linear regression was performed in the bvFTD group to determine correlation between specific cognitive test results, behavioural measures, and striatal volume. Covariates in these analyses comprised age, gender, education and intracranial volume.

Tests of normality (and distribution of residuals, collinearity) were performed to assess the suitability for the methods. Effect sizes in the ANCOVA analysis were expressed as partial Eta-squared. If a subject was unable to complete a particular cognitive test, they were excluded from the analysis.

Shape analysis was performed as follows. Using the 3-D object maps from ITK-SNAP, shape analysis was performed using the spherical harmonic toolkit SPHARM-PDM. Briefly, segmentations were minimally pre-processed to ensure a spherical topology. Subsequently the segmentations were described by spherical harmonic functions and then sampled into surfaces of 1002 points. These surfaces were then aligned using a rigid-body Procrustes alignment to a mean template created from the sample. Comparisons between groups were performed using MANCOVA utilising Hoteling T2-two sample statistic, and correlations using Pearson correlation coefficient. Correction for multiple comparisons were performed using a False Discovery Rate (FDR) estimation of 5% [34]. All analyses were normalised for head size using a scaling factor of (Mean ICV/ICV)^1/3^ [35, 36]. Demographic variables (age, gender and education) were included in a second run of the analyses to ensure that results were consistent with these covariates added. More details about this method are available in the S1 Text.

Statistical shape analysis provides visualizations of the local effect size via mean difference magnitude displacement maps and correlation maps. Mean difference displacement maps display the magnitude of surface change (deflation or inflation) in mm between corresponding points on the mean surfaces of patients with bvFTD relative to controls. Correlation maps display local correlation coefficients. Additionally, shape statistical analysis significance maps showing local statistical p-values, raw and corrected for FDR, are generated. For each structure a global measure of shape change was computed by averaging the displacement across the surface and using an ANOVA to compare the average displacement between groups. ICV was corrected as per SPHARM analyses.

## Results

### Demographics and Clinical Details

Demographic details are summarised in Table 1. As expected, there were significant differences in the MMSE score between the bvFTD group and healthy controls. There were no significant differences in age or gender distribution, but differences in educational level, with the bvFTD group having significantly fewer years of education. The bvFTD group had median illness duration of three years (ranging from one year to 12 years), median CDR of 1 (indicating mild dementia) and a median score on the FBI total score of 27.

One patient had a lacunar infarct in the right putamen, with this structure excluded from subsequent analysis.

### Groupwise and Severity Differences

#### Volumetric analysis—between groups

The bvFTD group had significantly smaller striatal structures than the control group (see Table 2). Specifically, analysis showed the bvFTD group had smaller caudate nuclei on the left (16% smaller, partial eta squared 0.173, p = 0.003) and right (11% smaller, partial eta squared 0.103, p = 0.023), as well as in the putamen on the left (18% smaller, partial eta squared 0.179, p = 0.002) and right (12% smaller, partial eta squared 0.081, p = 0.045) (Table 2).

**Table 2:** Striatal Volume Analysis—Groupwise difference and correlation with FBI scores.

Structure	Size difference in bvFTD group (%)	Effect Size (partial eta square)	Sig. (p)	Correlation with FBI 1–10		Correlation with FBI 11–22	
				Stand. Beta	Sig. (p)	Stand.Beta	Sig. (p)
**Left Caudate**	-16.055	0.173	0.003	-0.376	0.106	-0.194	0.378
**Right Caudate**	-11.898	0.103	0.023	-0.225	0.339	-0.219	0.312
**Left Putamen**	-18.189	0.179	0.002	-0.659	<0.001	-0.436	0.014
**Right Putamen**	-12.868	0.081	0.045	-0.455	0.035	-0.461	0.019

Groupwise differences assessed with ANCOVA. FBI correlations assessed with linear regression, with age, gender, education and intracranial volume as covariates.

#### Shape analysis—between-group and severity measures

**Fig 3:**
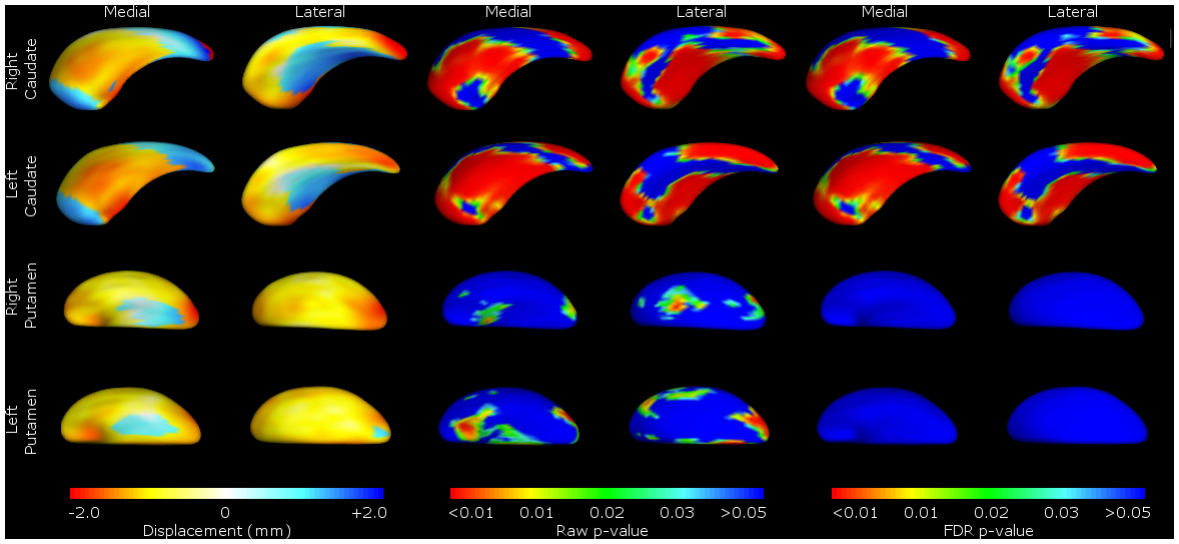
Shape analysis of bvFTD group vs. healthy controls. Shape change for FTLD patients relative to controls. For ease of reference, we present the data by structure (caudate or putamen), with medial and lateral views of the structure shown as labelled. These include the mean difference displacement maps (on the left) and *p*-value shape significance maps (middle and right). The displacement colour scale corresponds to the millimetres of deflation/inflation of the surface in that region; with warmer colours (such as red) corresponding to greater degrees of deflation, and cooler colours (such as blue) greater degrees of inflation. The *p*-value colour significance scale is identical for all images, and warmer colours refer to smaller *p*-values less than 0.05, with the blue colour corresponding to *p*-values above 0.05. Both ‘raw’ p-values and p-values corrected for False Discovery Rate (FDR) are shown.

Shape analysis of the bvFTD group revealed global shape deflation in the caudate bilaterally, most noticeably in the areas corresponding to the afferent connections from the dorsolateral prefrontal, orbitofrontal and mediofrontal/anterior cingulate cortex (Fig 3), with the left caudate in particular showing shape deflation in most areas of the structure. Measures of global shape change showed significant deflation in the right caudate (mean deflation 0.47mm, F(1,51) = 11.62, p = 0.00128) and in the left (mean deflation 0.60mm, F(1,51) = 18.22, p<0.0001), in line with the volumetric results.

Regarding the putamina, global shape change similarly reflected the volumetric results, with significant deflation in right putamen (mean deflation 0.76mm, F(1,50) = 11.9, p = 0.00115) and left (mean deflation 0.84mm, F(1,50) = 13.9, p = 0.000484). These were raw localised shape differences that did not survive correction for type II errors (FDR) (Fig 3).

**Fig 4:**
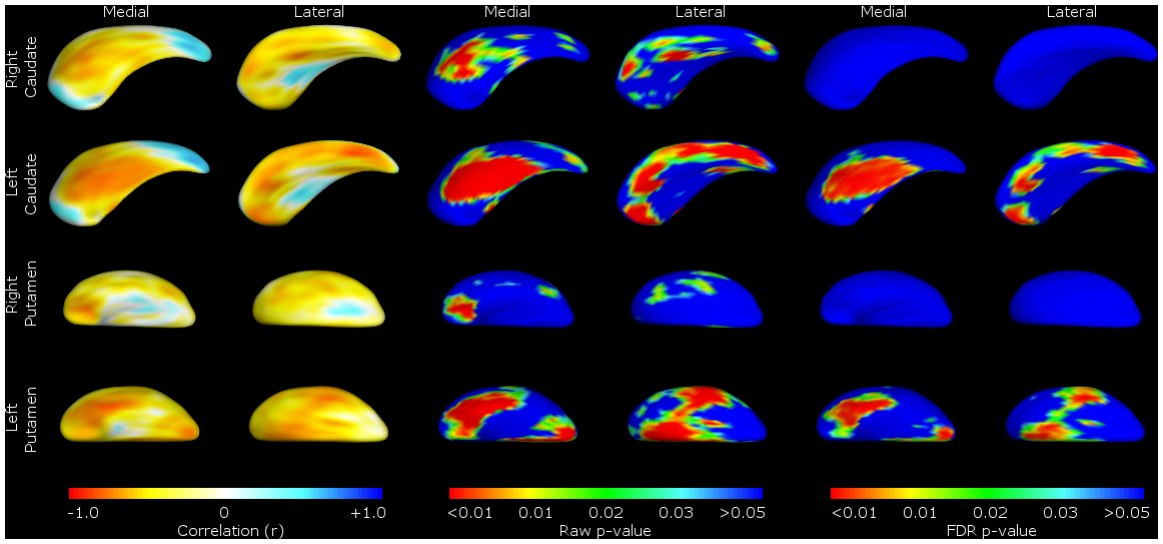
Striatal size correlated with a measure of disease severity—FTLD-CDR-SB. Shape change for correlations with Frontotemporal Lobar Degeneration Clinical Disease Rating—Sum of Boxes (FTLD-CDR-SB) in the bvFTD group only. For ease of reference, we present the data by structure (caudate or putamen), with medial and lateral views of the structure shown as labelled. These include the Pearson correlation coefficient colour maps (on the left) and *p*-value shape significance maps (middle and right). Pearson correlation coefficient colour maps are provided to visualise degree of positive and negative correlation of the surface in that region. Warmer colours (such as red) corresponding to negative correlation coefficients, and cooler colours (such as blue) to positive correlation coefficients. With the *p*-value colour significance scale, warmer colours refer to smaller *p*-values (less than 0.05), with the blue colour corresponding to *p*-values above 0.05. Both ‘raw’ p-values and p-values corrected for False Discovery Rate (FDR) are shown.

FTLD-CDR-SB scores showed a similar pattern, with worsening scores correlating with shape deflation in areas of the left caudate nucleus and putamen corresponding to the dorsolateral prefrontal mediofrontal/anterior cingulate and orbitofrontal cortex. There were no significant correlations with structures on the right (Fig 4).

### Correlation with Frontal Behavioural Inventory and other cognitive measures within the bvFTD group

#### Volumetric analyses—linear regression of behavioural and cognitive measures

Linear regression analysis examining striatal structures and the FBI scores for items 1–10, incorporating the covariates mentioned above, found significant negative correlations (i.e. higher the severity score, the smaller the striatal structure) with the volume of the putamen on the left (standardised beta = -0.659, p<0.001) and the right (standardised beta = -0.455, p = 0.035). Correlations with the ‘positive symptom’ section of the FBI (items 12–22) also showed a negative correlation with putamen volumes on the left (standardised beta = -0.436, p = 0.014) and the right (standardised beta = -0.461, p = 0.019). This correlation was not demonstrated with the caudate nuclei (Table 2).

The completion rates of the other cognitive tests and behavioural measures were significantly lower, with the Hayling B and Brixton, Color Word Interference and Trail-Making B scores only being completed by 12 of the 28 participants in the bvFTD group. No significant correlations were found between these measures and the volume or shape of any of the striatal structures.

#### Shape analyses—Pearson correlations of surface regions with behavioural and cognitive measures

**Fig 5:**
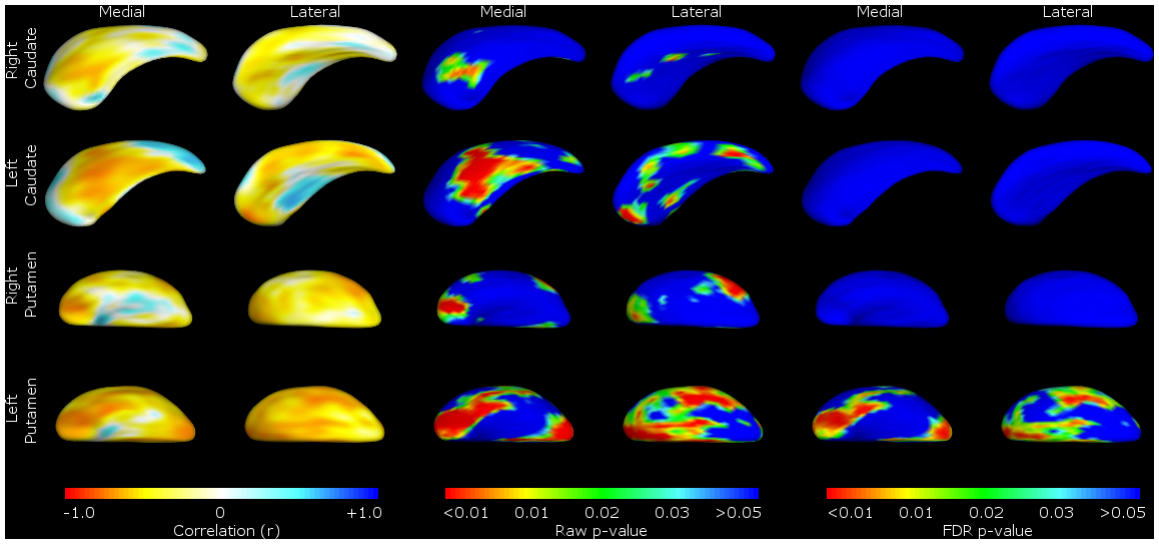
Shape Analysis of correlation between striatum and FBI 1–10 scores. Shape change for correlations with Frontal Behavioural Inventory (FBI) items 1–10 in the bvFTD group only. For ease of reference, we present the data by structure (caudate or putamen), with medial and lateral views of the structure shown as labelled. These include the Pearson correlation coefficient colour maps (on the left) and *p*-value shape significance maps (middle and right). Pearson correlation coefficient colour maps are provided to visualise degree of positive and negative correlation of the surface in that region. Warmer colours (such as red) corresponding to negative correlation coefficients, and cooler colours (such as blue) to positive correlation coefficients. With the *p*-value colour significance scale, warmer colours refer to smaller *p*-values (less than 0.05), with the blue colour corresponding to *p*-values above 0.05. Both ‘raw’ p-values and p-values corrected for False Discovery Rate (FDR) are shown.

**Fig 6:**
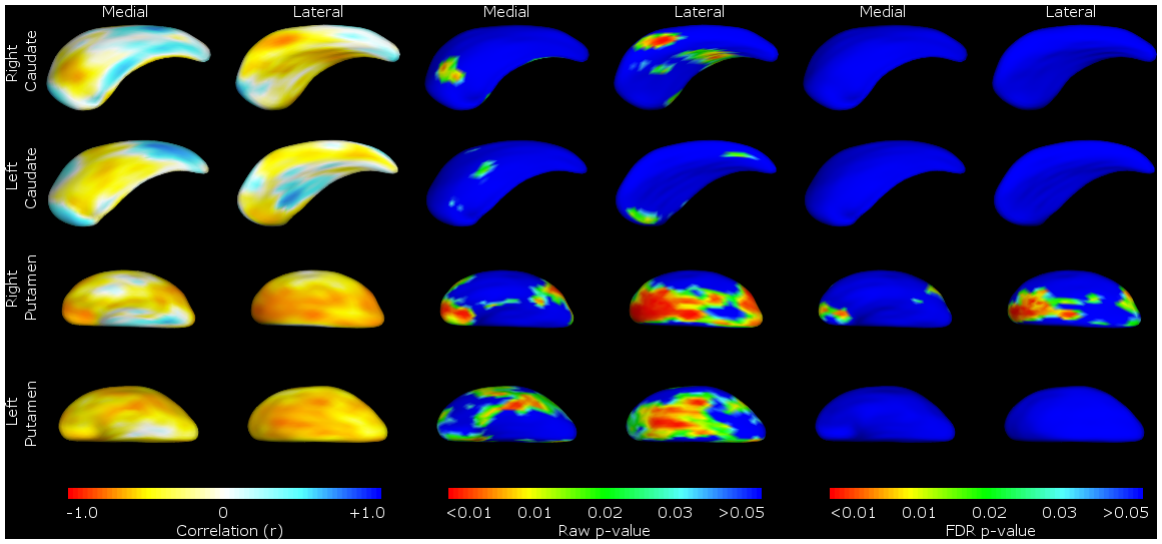
Shape Analysis of correlation between striatum and FBI 12–22 scores. Shape change for correlations with Frontal Behavioural Inventory (FBI) items 12–22 in the bvFTD group only. For ease of reference, we present the data by structure (caudate or putamen), with medial and lateral views of the structure shown as labelled. These include the Pearson correlation coefficient colour maps (on the left) and *p*-value shape significance maps (middle and right). Pearson correlation coefficient colour maps are provided to visualise degree of positive and negative correlation of the surface in that region. Warmer colours (such as red) corresponding to negative correlation coefficients, and cooler colours (such as blue) to positive correlation coefficients. With the *p*-value colour significance scale, warmer colours refer to smaller *p*-values (less than 0.05), with the blue colour corresponding to *p*-values above 0.05. Both ‘raw’ p-values and p-values corrected for False Discovery Rate (FDR) are shown.

In line with the volumetric changes, there was no significant localised shape change in the caudate associated with FBI scores. However, more significant global shape deflation was seen in the putamen—scoring on the FBI items 1–10 was associated with shape deflation in the left putamen (Fig 5), while higher scores on items 12–22 were associated with shape deflation on the right (Fig 6).

## Discussion

### Group comparisons: bvFTD versus controls

#### Volumetric analyses

This neuroimaging study of 28 subjects with FTLD and 26 age-matched healthy controls focused on the caudate nucleus and putamen, finding that the diagnosis of bvFTD was associated with significantly smaller bilateral caudate nuclei and putamina compared to controls.

#### Shape analyses

There were also significant localised shape deflation in many areas of the caudate nuclei (particularly those with afferent connections to the dorsolateral prefrontal, orbitofrontal and anterior cingulate cortex, as well as motor and oculomotor circuits—see Figs 1 and 2 [4, 37].

Though there were raw shape differences in bvFTD putaminal shape compared to controls, these did not survive type II error (FDR) correction, possibly due to inter-individual shape variability in the putamen (making it more difficult to segment) impacting in the context of the relatively small sample size, resulting in failure to reject a false null hypothesis.

The significant groupwise differences in the caudate nuclei in volume and shape are consistent with previous studies on bvFTD by our group on different FTLD datasets [10]. Confirming our previous findings for the caudate, the topography of the atrophy implicates virtually all cortico-striato-thalamic circuits. This result is similar to studies using voxel-based morphology (VBM) in bvFTD, which have found atrophy in caudate and putamen, as well as in the ventral striatum/nucleus accumbens [8]. Notably, we did not confirm our previous findings of considerable shape change in the putamen, although as noted above, volume changes were significant, in contradistinction to our previous papers where we found extensive atrophy in the putamen in the two different datasets [9, 10].

### Correlation of volume and shape with clinical features

#### Volumetric analyses

In addition to the groupwise analysis, the bvFTD subjects in this trial underwent a number of tests of executive functioning and behaviour that we investigated for correlation with volume and shape within the bvFTD group. Volumetric analysis demonstrated significant correlation between putamen volumes and scores on the FBI although these correlations were not found with caudate nucleus volumes. Tests of executive functioning had poor completion rates (less than half of the bvFTD group), and no significant correlation was found between striatal volumes and scores on these measures.

Interestingly the correlation with the FBI was with putaminal volumes rather than the caudate. The putamen also has significant afferent connections to the mediofrontal, dorsolateral prefrontal and orbitofrontal cortex regions, as well as its better-known links with prefrontal motor and motor circuits, and the correlation with measures of different aspects of the FBI is thus not surprising.

#### Shape analyses

In accordance with the correlations with volumes, shape analyses for behavioural disturbances within the bvFTD group highlight the putamina, with positive symptoms linked to the right putamen and negative to the left. Specifically, we found that correlations between striatal shape and overall (FTLD-CDR-SB) and negative behaviours (FBI 1–10: apathy, aspontaneity, indifference, inflexibility, personal neglect, disorganization, inattention, loss of insight, logopenia, verbal apraxia) of bvFTD were significant primarily on the left, correlated to most major fronto-putaminal circuits such as from the dorsolateral prefrontal, orbitofrontal, anterior cingulate and motor cortical regions. This indicates that deficit syndromes of bvFTD may have a structural basis in the putamen, discussed further below.

Within the bvFTD group, we found that FTLD-CDR-SB was significantly correlated with left caudate and putamen shape deflations, perhaps indicating a left-sided pathology relating to severity of presentation. The finding of correlation of FBI 1–10 negative symptom behaviour items to putamen shape may be a partial explanation for the correlation of severity of FTLD-CDR-SB, as these loss of function behaviours may specifically impact upon global severity of disease.

We found that positive behaviours (FBI 12–22) and negative behaviours (FBI 1–10) of bvFTD were not significantly correlated to caudate regions. However, as can be seen from the figures, there are small raw (unadjusted for multiple comparisons) shape correlations for these measure in caudate and putamen for the FBI and the subscales. Perhaps with larger sample sizes in future, we will be able to determine if there are perhaps weaker correlations extant.

Our results concord with other studies highlighting the contribution of the striatum for bvFTD behavioural symptomatology and, indirectly, to behavioural control in the healthy brain. Some studies have found association with changes in the nucleus accumbens [23, 38], and clinical measures of apathy in bvFTD have shown correlation with atrophy in the right caudate [39]. The putamen, particularly on the right, has been implicated in several studies: for instance, atrophy in the right putamen of bvFTD patients has been associated with increased scores on scales of apathy, disinhibition, aberrant eating and motor behaviours [40], reward-seeking behaviours (hypersexuality and sweet-food preference) [41] and binge eating [42, 43]. These studies provide evidence for the importance of the putamen, particularly the right putamen, in positive symptoms of behavioural disturbance (perseverations, excessive jocularity, poor judgement, inappropriateness, impulsivity and hyperorality), with negative symptoms of behavioural disturbance correlated with left putamen.

### Limitations

As a simple scalar summary value, the volume of a structure condenses much information on shape into a unitary measure: therefore, it is inherently a more reliable measure for both between-group and within-group analyses. Accordingly, it is expected that the findings with volumetric analyses may seem more robust. The shape of specifically topographically-linked structures such as the caudate and putamen is considered to yield additional information as the surface anatomy can be related to afferents from the cortex, albeit with a significant compression of inputs. However, the statistical analysis of shape involves multiple comparisons across many surface points and thus invokes a stringent adjustment for multiple comparisons for type I (permutation) and type II (FDR) errors, which may then discriminate against weaker or less robust raw shape findings. Another limitation of this study was the comparatively low power of the correlations associated with the executive functioning tests, with a large proportion of subjects unable to complete the majority of the testing, lowering the ability of any analysis to detect significant correlation with striatal structures. Finally, the caudate as compared to the putamen is more easily manually segmented due to the relatively clearer medial boundary with the ventricle, and hence may be the more reliable overall measurement.

## Conclusion

This study has confirmed the striatal volume and shape changes associated with a diagnosis of bvFTD compared to health controls seen in previous work from our group [9–11], but extends this by the demonstration of correlations between primarily putaminal size and shape deflation and bvFTD, both in behavioural measures and measures of clinical severity. This provides further clues to the involvement of different cortico-striato-thalamic networks in the symptomatology of bvFTD; in particular, that specific atrophy in the putamen may contribute to many of the central symptoms of the disorder. Furthermore, the correlation of left striatal shape with severity of dementia as measured by CDR suggests a dimensionality to the atrophic process. Together with the robust group differences, the correlation with clinical features and severity for the striatum, and especially the putamen for behavioural symptoms, indicate potential usefulness of shape and volume of the striatum as nascent biomarker *in vivo*, towards development of an ensemble of neuroimaging biomarkers.

Future directions include investigating the diagnostic value of striatal shape and volume data in clarifying the differential diagnosis of bvFTD, exploring correlational analysis with tractography of the cortico-striato-thlamic circuits, and incorporating functional magnetic resonance imaging data of intrinsic connectivity networks to integrate our findings with parallel neuroimaging research. In this way we can more precisely detect, map and track progression of bvFTD: spatially, temporally and in association with symptomatology.

## Supporting Information

S1 Text Computational Details of Spherical Harmonic Shape Analysis Group Comparisons, Error Corrections, Magnitude Displacement Maps. (DOCX)

## References

[1] Wittenberg D, Possin KL, Rascovsky K, Rankin KP, Miller BL, Kramer JH. The Early Neuropsychological and Behavioral Characteristics of Frontotemporal Dementia. Neuropsychol Rev. 2008;18(1):91–102. 10.1007/s11065-008-9056-z 18311522 PMC3000668

[2] Rascovsky K, Hodges JR, Knopman D, Mendez MF, Kramer JH, Neuhaus J, et al Sensitivity of revised diagnostic criteria for the behavioural variant of frontotemporal dementia. Brain. 2011;134(Pt 9):2456–77. 10.1093/brain/awr179 21810890 PMC3170532

[3] Mega MS, Cummings JL. Frontal-subcortical circuits and neuropsychiatric disorders. Jou Neuropsych Clin Neurosci. 1994;6:358–70. 10.1176/jnp.6.4.358 7841807

[4] Draganski B, Kherif F, Kloppel S, Cook PA, Alexander DC, Parker GJM, et al Evidence for Segregated and Integrative Connectivity Patterns in the Human Basal Ganglia. Jou Neurosci. 2008;28(28):7143–52. 10.1523/jneurosci.1486-08.2008 18614684 PMC6670486

[5] Looi JCL, Walterfang M, Velakoulis D, Macfarlane MD, Svensson LA, Wahlund LO. Frontotemporal dementia as a frontostriatal disorder: Neostriatal morphology as a biomarker and structural basis for an endophenotype. Aust N Z J Psychiatry. 2012;46(5):422–34. 10.1177/0004867411432076 22535292

[6] Alexander GE, DeLong MR, Strick PL. Parallel Organization of Functionally Segregated Circuits Linking Basal Ganglia and Cortex. Annu Rev Neurosci. 1986;9:357–81. 10.1146/annurev.ne.09.030186.002041 3085570

[7] Looi JCL, Walterfang M. Striatal morphology as a biomarker in neurodegenerative disease. Mol Psychiatry. 2012 10.1038/mp.2012.54 22584865

[8] Schroeter ML, Laird AR, Chwiesko C, Deuschl C, Schneider E, Bzdok D, et al Conceptualizing neuropsychiatric diseases with multimodal data-driven meta-analyses—the case of behavioral variant frontotemporal dementia. Cortex. 2014;57:22–37. 10.1016/j.cortex.2014.02.022 24763126 PMC4108513

[9] Looi JCL, Rajagopalan P, Walterfang M, Madsen SK, Thompson PM, Macfarlane MD, et al Differential putaminal morphology in Huntington's disease, frontotemporal dementia and Alzheimer's disease. Aust N Z Jou Psychiatry. 2012;46(12):1145–58. 10.1177/0004867412457224 22990433 PMC4113021

[10] Looi JCL, Walterfang M, Styner M, Niethammer M, Svensson LA, Lindberg O, et al Shape analysis of the neostriatum in subtypes of frontotemporal lobar degeneration: Neuroanatomically significant regional morphologic change. Psychiatry Res: Neuroimaging. 2011;191(2):98–111. 10.1016/j.pscychresns.2010.09.014 21237621

[11] Looi JCL, Lindberg O, Zandbelt BB, Ostberg P, Andersen C, Botes L, et al Caudate Nucleus Volumes in Frontotemporal Lobar Degeneration: Differential Atrophy in Subtypes. AJNR A J Neuroradiol. 2008;29(8):1537–43. 10.3174/ajnr.A1168 18782907 PMC8119022

[12] Looi JCL, Svensson L, Lindberg O, Zandbelt BB, Ostberg P, Orndahl E, et al Putaminal Volume in Frontotemporal Lobar Degeneration and Alzheimer Disease: Differential Volumes in Dementia Subtypes and Controls. AJNR A J Neuroradiol. 2009;30(8):1552–60. 10.3174/ajnr.A1640 19497964 PMC7051615

[13] Kim EJ, Rabinovici GD, Seeley WW, Halabi C, Shu H, Weiner MW, et al Patterns of MRI atrophy in tau positive and ubiquitin positive frontotemporal lobar degeneration. J Neurol Neurosurg Psychiatry. 2007;78(12):1375–8. 10.1136/jnnp.2006.114231 17615169 PMC2095621

[14] Josephs KA, Whitwell JL, Parisi JE, Petersen RC, Boeve BF, Jack CR Jr., et al Caudate atrophy on MRI is a characteristic feature of FTLD-FUS. Eur J Neurol. 2010;17(7):969–75. 10.1111/j.1468-1331.2010.02975.x 20236174 PMC2989679

[15] Seeley WW, Crawford RK, Zhou J, Miller BL, Greicius MD. Neurodegenerative Diseases Target Large-Scale Human Brain Networks. Neuron. 2009;62(1):42–52. 10.1016/j.neuron.2009.03.024 19376066 PMC2691647

[16] Farb NAS, Grady CL, Strother S, Tang-Wai DF, Masellis M, Black S, et al Abnormal network connectivity in frontotemporal dementia: evidence for prefrontal isolation. Cortex. 2013;49(7):1856–73. 10.1016/j.cortex.2012.09.008 23092697

[17] Grahn JA, Parkinson JA, Owen AM. The cognitive functions of the caudate nucleus. Prog Neurobiol. 2008;86(3):141–55. 10.1016/j.pneurobio.2008.09.004 . 18824075

[18] Macfarlane MD, Looi JCL, Walterfang M, Spulber G, Velakoulis D, Crisby M, et al Executive dysfunction correlates with caudate nucleus atrophy in patients with white matter changes on MRI: A subset of LADIS. Psychiatry Res: Neuroimaging. 2013;214(1):16–23. 10.1016/j.pscychresns.2013.05.010 23916538

[19] Santillo AF, Martensson J, Lindberg O, Nilsson M, Manzouri A, Landqvist Waldo M, et al Diffusion tensor tractography versus volumetric imaging in the diagnosis of behavioral variant frontotemporal dementia. PLoS One. 2013;8(7):e66932 10.1371/journal.pone.0066932 23874403 PMC3715470

[20] Blennow K, Hampel H, Weiner M, Zetterberg H. Cerebrospinal fluid and plasma biomarkers in Alzheimer disease. Nat Rev Neurol. 2010;6(3):131–44. Available: http://www.nature.com/nrneurol/journal/v6/n3/suppinfo/nrneurol.2010.4_S1.html. 10.1038/nrneurol.2010.4 20157306

[21] Kertesz A, Davidson W, Fox H. Frontal behavioral inventory: Diagnostic criteria for frontal lobe dementia. Can J Neurol Sci. 1997;24:29–36. 9043744 10.1017/s0317167100021053

[22] Zamboni G, Huey ED, Krueger F, Nichelli PF, Grafman J. Apathy and disinhibition in frontotemporal dementia. Neurology. 2008;71(10):736–42. 10.1212/01.wnl.0000324920.96835.95 18765649 PMC2676948

[23] Franceschi M, Anchisi D, Pelati O, Zuffi M, Matarrese M, Moresco RM, et al Glucose metabolism and serotonin receptors in the frontotemporal lobe degeneration. Ann Neurol. 2005;57(2):216–25. 10.1002/ana.20365 . 15668960

[24] Grahn JA, Parkinson JA, Owen AM. The role of the basal ganglia in learning and memory: Neuropsychological studies. Behav Brain Res. 2009;199(1):53–60. 10.1016/j.bbr.2008.11.020 19059285

[25] Tekin S, Cummings JL. Frontal—subcortical neuronal circuits and clinical neuropsychiatry: An update. J Psychosom Res. 2002;53(2):647–54. 10.1016/S0022-3999(02)00428-2 12169339

[26] Delis DC, Kaplan E, Kramer JH. Delis Kaplan executive function system (D-KEFS): Examiner's manual. San Antonio: The Psychological Coroporation; 2001.

[27] Burgess P, Shallice T. The Hayling and Brixton tests. Thurstone Suffolk: Pearson; 1997.

[28] Reitan RM. Trail Making Test: Manual for Aministration, Scoring and Interpretation. Indianapolis: Indiana University Medical Center; 1958.

[29] Tombaugh T. Trail Making Test A and B: Normative data stratified by age and education. Arch Clin Neuropsychol. 2004;19:203–14. 15010086 10.1016/S0887-6177(03)00039-8

[30] Yushkevich PA, Piven J, Hazlett HC, Smith RG, Ho S, Gee JC, et al User-guided 3D active contour segmentation of anatomical structures: significantly improved efficiency and reliability. NeuroImage. 2006;13(3):1116–28. 16545965 10.1016/j.neuroimage.2006.01.015

[31] Looi J, Lindberg O, Liberg B, Tatham V, Kumar R, Maller J, et al Volumetrics of the caudate nucleus: Reliability and validity of a new manual tracing protocol. Psychiatry Res: Neuroimaging. 2008;163(3):279–88. 10.1016/j.pscychresns.2007.07.005 18657402

[32] ENIGMA-Consortium. Genome-Wide Association Meta-Analysis of Hippocampal Volume via the ENIGMA Consortium. Organisation for Human Brain Mapping 2011 Meeting2011.

[33] Buckner RL, Head D, Parker J, Fotenos AF, Marcus D, Morris JC, et al A unified approach for morphometric and functional data analysis in young, old, and demented adults using automated atlas-based head size normalization: reliability and validation against manual measurement of total intracranial volume. NeuroImage. 2004;23(2):724–38. 10.1016/j.neuroimage.2004.06.018 15488422

[34] Genovese CR, Lazar NA, Nichols T. Thresholding of statistical maps in functional neuroimaging using the false discovery rate. NeuroImage. 2002;15:870–8. 11906227 10.1006/nimg.2001.1037

[35] Styner M, Oguz I, Xu S, Brechbuhler C, Pantazis D, Levitt JJ, et al Framework for the statistical shape analysis of brain structures using SPHARM-PDM. Insight Journal. 2006;1:1–21. PMC3062073 21941375

[36] Styner M, Oguz I, Xu S, Pantazis D, Gerig G. Statistical group differences in anatomical shape analysis using the Hotelling T2 metric. Proc SPIE 6512, Medical, Imaging 2007:65123, z1–z11.

[37] Haber S. The primate basal ganglia: parallel and integrative networks. J Chem Neuroanat. 2003;26(4):317–30. 10.1016/j.jchemneu.2003.10.003 14729134

[38] Zamboni G, Huey ED, Krueger F, Nichelli PF, Grafman J. Apathy and disinhibition in frontotemporal dementia: Insights into their neural correlates. Neurology. 2008;71:736–42. 10.1212/01.wnl.0000324920.96835.95 18765649 PMC2676948

[39] Eslinger PJ, Moore P, Antani S, Anderson C, Grossman M. Apathy in frontotemporal dementia: Behavioral and neuroimaging correlates. Behav Neurol. 2012;25(2):127–36. 10.3233/BEN-2011-0351 22425723 PMC3640327

[40] Halabi CBS, Halabi ABS, Dean DLBS, Wang PN, Boxer AL, Trojanowski JQ, et al Patterns of Striatal Degeneration in Frontotemporal Dementia. Alzheimer Dis Assoc Disord. Jan-Mar 2013. 2013;27(1):74–83. 10.1097/WAD.0b013e31824a7df4 22367382 PMC3389579

[41] Perry DC, Sturm VE, Seeley WW, Miller BL, Kramer JH, Rosen HJ. Anatomical correlates of reward-seeking behaviours in behavioural variant frontotemporal dementia. Brain. 2014;137(6):1621–6. 10.1093/brain/awu075 24740987 PMC4032100

[42] Woolley JD, Gorno-Tempini ML, Seeley WW, Rankin K, Lee SS, Matthews BR, et al Binge eating is associated with right orbitofrontal-insular-striatal atrophy in frontotemporal dementia. Neurology. 2007;69(14):1424–33. 10.1212/01.wnl.0000277461.06713.23 17909155

[43] Garibotto V, Borroni B, Agosti C, Premi E, Alberici A, Eickhoff SB, et al Subcortical and deep cortical atrophy in Frontotemporal Lobar Degeneration. Neurobiol Aging. 2011;32(5):875–84. 10.1016/j.neurobiolaging.2009.05.004 19501427

