# Supplementary material for: Striatal Atrophy in the Behavioural Variant of Frontotemporal Dementia: Correlation with Diagnosis, Negative Symptoms and Disease Severity
Source: PLoS One. 2015 Jun 15;10(6):e0129692. doi: 10.1371/journal.pone.0129692 (PMC4468218; doi:10.1371/journal.pone.0129692)
Supplement: S1 Text — (DOCX) [file pone.0129692.s001.docx]

**Supplementary Material 1. Computational Details of Spherical Harmonic Shape Analysis Group Comparisons, Error Corrections, Magnitude Displacement Maps**

(Reproduced/adapted from Styner et al., 2006)

**Group comparisons of shape**

We calculate group differences by analyzing the spatial location of each point. For this option, no template is necessary and multivariate statistics of the (*x*,*y*,*z*) location is necessary. We have chosen to use the Hotelling *T* ^2^ two sample difference metric as a measurement of how 2 groups locally differ from each other. The standard Hotelling *T* ^2^ is defined as *T* ^2^ = (*μ*1− *μ*2)' (Σ ( 1 /*n*1 + 1 /*n*2))^−1^(*μ*1− *μ*2), where Σ = (Σ_1_(*n*1− 1) + Σ_2_(*n*2− 1))/(*n*1+ *n*2− 2) is the pooled covariance matrix. An alternative modified Hotelling *T* ^2^ metric is less sensitive to group differences of the covariance matrixes and the number of samples(Styner et al., 2007): *T* 2 = (*μ*1− *μ*2)'(Σ_1_ 1 /*n*1 + Σ_2_ 1 /*n*2)^−1^(*μ*1− *μ*2). All our current studies are based on this modified Hotelling *T* ^2^ metric.

We then want to test the two groups for differences in the means of the selected difference metric (univariate: Student t, multivariate: Hotelling *T* ^2^) at each spatial location. Permutation tests are a valid and tractable approach for such an application, as they rely on minimal assumptions and can be applied even when the assumptions of the parametric approach are untenable. Non-parametric permutation tests are exact, distribution free and adaptive to underlying correlation patterns in the data. Further, they are conceptually straightforward and, with recent improvements in computing power, are computationally tractable.

Our null hypothesis is that the distribution of the locations at each spatial element is the same for every subject regardless of the group. Permutations among the two groups satisfy the exchangeability condition, i.e. they leave the distribution of the statistic of interest unaltered under the null hypothesis. Given n1 members of the first group a_k_, k = 1 . . . n1 and n2 members of the second group b_k_, k = 1 . . . n2, we can create M ≤((n1 + n2)!)/(n2!) permutation samples. A value of M from 20000 and up should yield results that are negligibly different from using all permutations.

**Corrections for multiple comparisons**

In this study, we are employing non-parametric permutation tests and false discovery rate as two alternative correction methods for the multiple comparison problem.

**Correction for Type I Errors**

The correction method for multiple comparisons is based on computing first the local p-values using permutation tests. The minimum of these p-values across the surface is then computed for every permutation. The appropriate corrected p-value at level α can then be obtained by the computing the value at the α-quantile in the histogram of these minimum values. Using the minimum statistic of the p-values, this method correctly controls for the family wise error rate, or the false positives, but no control of the false negatives is provided. The resulting corrected local significance values can thus be regarded as pessimistic estimates akin to a simple Bonferroni correction.

**Correction for Type II Errors**

Additionally to the non-parametric permutation correction, we have also implemented and applied a False Discovery Rate Estimation (FDR) method. The innovation of this procedure is that it controls the expected proportion of false positives only among those tests for which a local significance has been detected. The FDR method thus allows an expected proportion (usually 5%) of the FDR corrected significance values to be falsely positive. The correction using FDR provides an interpretable and adaptive criterion with higher power than the non-parametric permutation tests. FDR is further simple to implement and computationally efficient even for large datasets.

The FDR correction is computed as follows:

1. Select the desired FDR bound q, e.g. 5%. This is the maximum proportion of false positives among the significant tests that you are willing to tolerate (on average).

2. Sort the p-values smallest to largest.

3. Let p_q_ be the p-value for the largest index *i* of the sorted p-values p_sort,i_ ≤ q·i/N, where N is the number of vertices.

4. Declare all locations with a p-value p ≤ p_q_ significant.

**Mean difference magnitude difference maps**

These are calculated as the map of the absolute difference in the mean surfaces between groups (based upon the computations above), derived from the lengths of the difference vectors (that is the difference in vectors for analogous surface points between the groups).

**Correlation maps**

These are calculated via correlating an independent variable with the length of the mean distance vector at each surface location, producing a signed Spearman’s rank correlation co-efficient for each point.

**References**

Styner, M., Oguz, I., Xu, S., Brechbuhler, C., Pantazis, D., Levitt, J.J., Shenton, M.E., Gerig, G. 2006. Framework for the statistical shape analysis of brain structures using

SPHARM-PDM. Insight J. 1–21.

Styner, M., Oguz, I., Xu, S., Pantazis, D., Gerig, G., 2007. Statistical group differences in anatomical shape analysis using the Hotelling T2 metric. Proc SPIE 6512, Medical Imaging 2007, pp 65123, z1-z11.
